# Complete Genome Sequence of *Mycolicibacterium hassiacum* DSM 44199

**DOI:** 10.1128/MRA.01522-18

**Published:** 2019-01-24

**Authors:** Mercedes Sánchez, Alba Blesa, Eva Sacristán-Horcajada, José Berenguer

**Affiliations:** a Centro de Biología Molecular Severo Ochoa, Universidad Autónoma de Madrid-Consejo Superior de Investigaciones Científicas, Madrid, Spain; b Universidad Francisco de Vitoria, Madrid, Spain; Queens College

## Abstract

Mycolicibacterium hassiacum is the most thermophilic of all the mycobacteria. A partial sequence based on Illumina technology of around 5 Mbp was published in 2012. Here, we report the 5,269,097-bp complete genome sequence assembled into a single circular chromosome.

## Abstract (precis)

Mycolicibacterium hassiacum is the most thermophilic of all the mycobacteria. A partial sequence based on Illumina technology of around 5 Mbp was published in 2012.

## ANNOUNCEMENT

Mycolicibacterium hassiacum (1), described in 1997 as Mycobacterium hassiacum (2), is characterized by its ability to grow at temperatures up to 65°C (2). Its fast growth and cell yield and the conservation and thermostability of most genus-specific enzymes (3, 4) make this strain an attractive laboratory mycobacterial model. Also, its isolation from clinical samples supports the conservation of virulence traits of its pathogenic relatives (5). Mycolicibacterium hassiacum DSM 44199 was donated by N. Empadinhas, whose group carried out a partial sequencing of the strain (6). In that work, the sequence was divided into 169 contigs, with an estimated genome size of 5 Mbp. Here, we present the whole-genome sequence obtained by PacBio technology, with further refinement using original Illumina reads. The strain was grown at 50°C under stirring (200 rpm) in Middlebrook 7H9 broth (BD Difco) with 0.15% (vol/vol) Tween 80 (Sigma-Aldrich) and 0.2% (vol/vol) glycerol as a carbon source. The cells grown up to an optical density at 600 nm (OD_600_) of 0.8 were harvested and washed by centrifugation, and the total DNA was isolated by a combination of mechanical breaking with glass beads (Sigma-Aldrich) and phenol-chloroform extraction (7). Twenty-kilobase DNA fragments were selected using BluePippin equipment (Sage Science), and a library was prepared according to Pacific Biosciences protocols. The Pacific Biosciences RS II equipment with P6-C4 chemistry (polymerase attachment by binding P6 kit) was used, with a recording time of 360 min.

A total of 69,152 reads, with an average length of 17,024 bp and a mean coverage of 223-fold, were obtained. The HGAP version 3 software (SMRT Analysis software version 2.3.0; Pacific Biosciences) was used with its default settings for the assembly. This produced a single linear contig of 5,282,087 bp. Its circularization was carried out using the Minimus2 software and further corrected with the RS_Resequencing.1 software (SMRT Analysis version 2.3.0). A single circular contig of 5,268,611 bp was obtained after this procedure.

The PROKKA software version 1.12 (8) was employed on this circular chromosome for automatic coding sequence (CDS) annotation, with parameters set for gene code 11 and using Mycobacterium genus-specific databases for assignment of putative function. A manual inspection of the annotation obtained revealed that many conserved proteins within the Mycobacterium genus appeared to be truncated due to the inclusion of an additional base in some homopolymers of the assembled genome, including proteins essential for viability, such as the β′-subunit of the RNA polymerase, the chromosome partition protein Smc, the FtsK ATPase involved in DNA segregation, and the RecO protein involved in DNA repair. Due to this, the PacBio-based sequence was further refined by using the Illumina reads available online (6) using the PacBio-utilities and Pilon (version 1.22) programs (9), configured to fix only the indels detected. This allowed us to correct 483 positions in the sequence assembled, corresponding to homopolymers for which PacBio reads included an additional base. Once corrected, the initially truncated essential genes were translated to proteins of the size and sequence homology expected for their homologues. The final genome of 5,269,097 bp encodes 5,333 putative proteins, 53 tRNAs, and 2 rRNA clusters. The mean GC content was 69.29% (34.55% Cs and 34.74% Gs), with a coding density of 91.5%.

The KEGG Automatic Annotation Server and its internal BlastKOALA annotation tool (10) led to the identification of 236 pathways, of which 61 contained more than 10 genes, including 20 genes putatively involved in the metabolism of steroids (KEGG steroid degradation pathway 00984). As this bacterium metabolizes different kinds of sterols from the environment, the identification of enzymes of this pathway constitutes a first step toward the isolation of specific mutants for relevant biotechnological bioconversions involving these molecules.

### Data availability.

The raw reads and the annotated assembly of the whole genome of Mycolicibacterium hassiacum have been deposited in NCBI under the accession number GCA_900603025.
